# Supplementary figures and images for: Associating ridesourcing with road safety outcomes: Insights from Austin, Texas
Source: PLoS One. 2021 Mar 18;16(3):e0248311. doi: 10.1371/journal.pone.0248311 (PMC7971567; doi:10.1371/journal.pone.0248311)

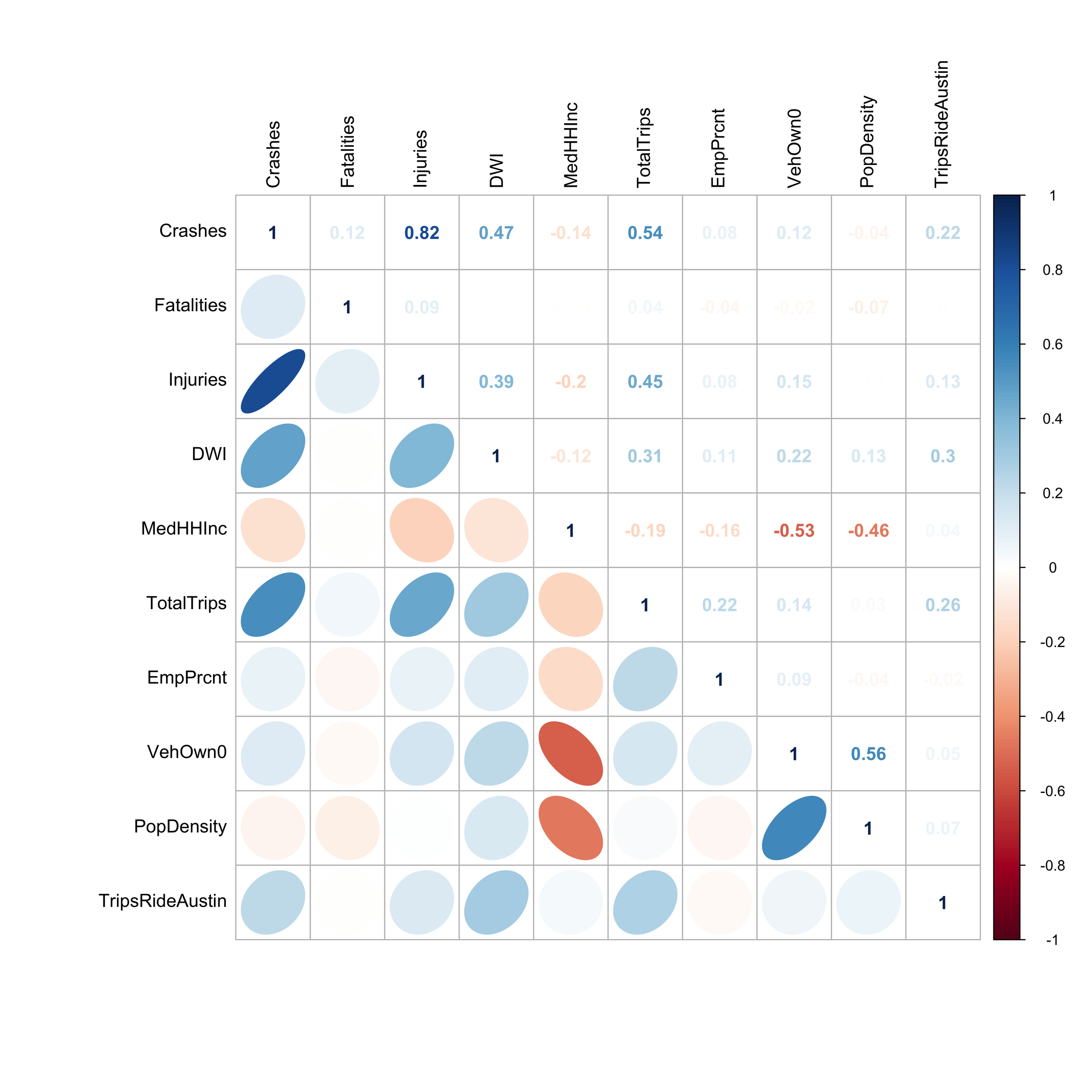

Supplement: S1 Fig — The correlation matrix results of the four safety outcome variables, the ridesourcing use, and control variables are presented to showcase associations between those. (TIF) [file pone.0248311.s001.tif]
