# Supplementary material for: Associating ridesourcing with road safety outcomes: Insights from Austin, Texas
Source: PLoS One. 2021 Mar 18;16(3):e0248311. doi: 10.1371/journal.pone.0248311 (PMC7971567; doi:10.1371/journal.pone.0248311)
Supplement: S1 Appendix — (PDF) [file pone.0248311.s003.pdf]

S1 Appendix. Traffic time series analysis. Due to unavailability of OD traffic data for each census tract in Travis County for the whole period of the analysis (January 2012-June 2014 and June 2016-April 2017), a time series, autoregressive integrated moving average model was fitted. We have monthly OD trips rate output data from the StreetLight Data platform from January 2016 until December 2018 [47]. The ridesourcing trip rates refer to both origins and destinations for trips that start and end at different census tracts. If both trip's origin and destination fall within the same census tract then it is only counted once. We apply the time series model with a transfer function (based on historical gasoline prices) to populate the historical OD trips data for every spatial unit of analysis. The autoregressive order of the model ARIMA(p, q, d) dictates the number of the lagged values $x_{i,t-1}$ that have an impact on $x_{i,t}$ OD trips. The order of the moving average direction q is the number of the lagged error terms used in the model, and d is the fractional integration parameter used to force stationarity. In our analysis $p = 1, q = 0$, and $d = 1$. The transfer function is the average monthly $ per gallon gas price. The chosen model was based on comparisons of R squared adjusted and Akaike's Information Criterion. Its results are used to populate the OD Trips data fields of this analysis.
