# Supplementary material for: Associating ridesourcing with road safety outcomes: Insights from Austin, Texas
Source: PLoS One. 2021 Mar 18;16(3):e0248311. doi: 10.1371/journal.pone.0248311 (PMC7971567; doi:10.1371/journal.pone.0248311)
Supplement: S1 Table — (PDF) [file pone.0248311.s004.pdf]

S1 Table. Robustness check using shorter RideAustin operational period dataset (October 2016-March 2017): spatial lag fixed-effects model results.

	**Log(1+Crashes)**<br>β		**Log(1+Injuries)**<br>β		**Log(1+Fatalities)**<br>β		**Log(1+DWI)**<br>β	
**Percent of employment**	-0.042<br>[0.179]		0.448<br>[0.242]		-0.096<br>[0.057]		0.051<br>[0.171]	
**Median HH income**	-2.4910^{-6}<br>[1.1310^{-6}]	**.**	-2.5710^{-6}<br>[1.5410^{-6}]		0.3310^{-6}<br>[0.3610^{-6}]		-1.0910^{-6}<br>[1.0910^{-6}]	
**Percent of zero vehicle ownership**	-0.689<br>[0.342]	**.**	-0.675<br>[0.461]		0.145<br>[0.101]		-0.114<br>[0.328]	
**Population density**	2.5210^{-6}<br>[1.4810^{-6}]		2.5410^{-6}<br>[2.0010^{-6}]		-1.2410^{-6}<br>[0.4710^{-6}]	**	1.6410^{-6}<br>[1.4210^{-6}]	
**OD trips**	7.7810^{-7}<br>[7.1810^{-7}]		1.4510^{-6}<br>[0.9710^{-6}]		0.1610^{-6}<br>[0.2310^{-6}]		1.2410^{-6}<br>[0.6910^{-6}]	
**Log(1+trips RideAustin)**	-0.011<br>[0.008]		-0.028<br>[0.014]	*	-0.002<br>[0.003]		-0.029<br>[0.008]	**
λ	0.101<br>[0.018]	***	0.005<br>[0.019]	*	0.008<br>[0.019]		0.042<br>[0.019]	*
**LM test (df=1)**	29.35	***	8.71	*	0.19		4.85	**.**

Symbol *** corresponds to $p< 0.0001$, ** to $p< 0.001$, * to $p< 0.01$, and **.** to $p< 0.05$.
