# Supplementary material for: Associating ridesourcing with road safety outcomes: Insights from Austin, Texas
Source: PLoS One. 2021 Mar 18;16(3):e0248311. doi: 10.1371/journal.pone.0248311 (PMC7971567; doi:10.1371/journal.pone.0248311)
Supplement: S2 Table — (PDF) [file pone.0248311.s005.pdf]

S2 Table. Robustness check using shorter RideAustin operational period dataset (October 2016-March 2017): spatial error fixed-effects model results.

	**Log(1+Crashes)** β		**Log(1+Injuries)** β		**Log(1+Fatalities)** β		**Log(1+DWI)** β	
**Percent of employment**	0.054 [0.179]		0.453 [0.241]		-0.096 [0.057]		0.053 [0.172]	
**Median HH income**	-2.4510^{-6} [1.1410^{-6}]	.	-2.5510^{-6} [1.5310^{-6}]		0.3410^{-6} [0.3610^{-6}]	.	-1.0610^{-6} [1.0910^{-6}]	
**Percent of zero vehicle ownership**	-0.655 [0.344]		-0.675 [0.464]		0.146 [0.109]		-0.106 [0.329]	
**Population density**	2.4110^{-6} [1.4810^{-6}]		2.4110^{-6} [2.0010^{-6}]		-1.2410^{-6} [0.4710^{-6}]	**	1.6310^{-6} [1.4210^{-6}]	
**OD trips**	7.8310^{-7} [7.4110^{-7}]		1.4510^{-6} [0.9810^{-6}]		0.1610^{-6} [0.2310^{-6}]		1.2410^{-6} [0.6910^{-6}]	
**Log(1+trips RideAustin)**	-0.011 [0.009]		-0.028 [0.012]	*	-0.002 [0.002]		-0.029 [0.008]	**
ρ	0.100 [0.018]	***	0.055 [0.019]	**	0.008 [0.019]		0.041 [0.019]	.
**LM test (df=1)**	28.54	***	8.52	.	0.19		4.85	.

Symbol *** corresponds to $p < 0.0001$, ** to $p < 0.001$, * to $p < 0.01$, and . to $p < 0.05$.
