# Supplementary material for: Associating ridesourcing with road safety outcomes: Insights from Austin, Texas
Source: PLoS One. 2021 Mar 18;16(3):e0248311. doi: 10.1371/journal.pone.0248311 (PMC7971567; doi:10.1371/journal.pone.0248311)
Supplement: S3 Table — (PDF) [file pone.0248311.s006.pdf]

S3 Table. Robustness check using shorter RideAustin operational period dataset (October 2016-March 2017): SARAR model results.

	**Log(1+Crashes)**<br>β		**Log(1+Injuries)**<br>β		**Log(1+Fatalities)**<br>β		**Log(1+DWI)**<br>β	
**Percent of employment**	-0.019<br>[0.175]		0.438<br>[0.241]		-0.092<br>[0.056]		0.051<br>[0.172]	
**Median HH income**	-2.5210^{-6}<br>[1.1210^{-6}]	.	-2.6010^{-6}<br>[1.5310^{-6}]		0.3810^{-6}<br>[0.3610^{-6}]		-1.1010^{-6}<br>[1.0910^{-6}]	
**Percent of zero vehicle ownership**	-0.743<br>[0.329]	.	-0.703<br>[0.456]		0.151<br>[0.110]		-0.118<br>[0.327]	
**Population density**	2.7310^{-6}<br>[1.4610^{-6}]	.	2.7610^{-6}<br>[1.9910^{-6}]		-1.2810^{-6}<br>[0.4610^{-6}]	**	1.6410^{-6}<br>[1.4210^{-6}]	
**OD Trips**	0.6410^{-6}<br>[0.6610^{-6}]		1.4210^{-6}<br>[0.9410^{-6}]		0.1810^{-6}<br>[0.2410−6]		1.2210^{-6}<br>[0.7210^{-6}]	.
**Log(1+trips RideAustin)**	-0.009<br>[0.007]		-0.026<br>[0.011]	*	-0.002<br>[0.003]		-0.028<br>[0.011]	**
λ	0.307<br>[0.076]	***	0.154<br>[0.171]		-0.284<br>[0.096]	*	0.059<br>[0.418]	
ρ	-0.236<br>[0.094]	.	-0.105<br>[0.188]		0.267<br>[0.079]	***	-0.017<br>[0.043]	
**LM: lag (df=1)**	7.54	*	1.04		1.16		3.35	
**LM: error (df=1)**	6.73	*	0.86		1.19		3.08	
**Hausman test (df=6) chi-squared**	139.51	***	92.48	***	7.60	.	121.85	***

Symbol *** corresponds to $p < 0.0001$, ** to $p < 0.001$, * to $p < 0.01$, and . to $p < 0.05$.
